# Next-generation sequencing identified novel truncating mutations in BBS9 causing Bardet Biedl syndrome in two Iranian consanguineous families

**DOI:** 10.22037/ijcn.v16i1.31650

**Published:** 2022-01-01

**Authors:** Susan AKBAROGHLI, Daniz KOOSHAVAR, Zahra GOLCHEHRE, Arezou KARAMZADE, Mohammad SABERI, Mohammad Reza ALAEI, Masoud ABBASI SADEGH, Mostafa ASADOLLAHI, Mohammad KERAMATIPOUR

**Affiliations:** 1 Department of Pediatrics, Mofid Children’s Hospital, Faculty of Medicine, Shahid Beheshti University of Medical Sciences, Tehran, Iran; 2 Department of Medical Genetics, Faculty of Medicine, Tehran University of Medical Sciences, Tehran, Iran; 3 These authors contributed equally to this work and are considered as co-first-authors.

**Keywords:** Bardet-Biedl syndrome, High-Throughput Nucleotide Sequencing, *BBS9*, Iran

## Abstract

**Objectives:**

Bardet-Biedl syndrome (BBS) is an autosomal recessive pleiotropic ciliopathy, which includes multi-organ clinical manifestations. The known genes involved in the development of the disease account for the causality in about 80% of the examined cases.

**Materials & Methods:**

We investigated two Iranian unrelated clinically diagnosed BBS patients, using a targeted next-generation sequencing panel consisting of 18 known BBS genes. The detected variants were investigated in the pedigree and studied using *in silico* tools for their pathogenicity. Patients’ phenotypes were also assessed.

**Results:**

Novel homozygous variants were detected in *BBS9* gene in each patient, c.2014C>T, p.Gln672Ter and c.673_674insAA, p.Gln225GlnfsX10. The variants were segregated in the corresponding pedigree and were authenticated to obtain enough evidence to be categorized as pathogenic variants.

**Conclusion:**

Patients with truncating mutations in the same gene seem to show similar phenotypic features. Detection of novel and family-specific mutations is typically expected in the genetic hereditary diseases in Iran, which can finally lead to prevent the recurrence of the disease in the consanguineous marriages

## Introduction

Bardet-Biedl syndrome (BBS) is one of the most pleiotropic ciliopathies, which is mostly considered to be inherited in an autosomal recessive pattern; however, there is evidence that BBS presents with an oligogenic nature (1, 2). BBS shows interfamilial and intrafamilial variations in its multi-organ clinical manifestations (3, 4). It is characterized by rod­cone dystrophy, truncal (central) obesity, postaxial polydactyly, learning disabilities, hypogenitalism, and renal abnormalities as primary features. Secondary features include speech/developmental delay, non-retinal eye abnormalities, brachydactyly/syndactyly, facial dysmorphism, ataxia, behavioral traits, hepatic fibrosis, diabetes mellitus, oro-dental abnormalities, cardiovascular anomalies, Hirschsprung’s disease, anosmia, endocrine gland disturbances, and hearing loss. These features may partly be found in BBS patients (3, 5-8). Clinical manifestations are diagnostic for BBS when four primary features or three primary plus at least two secondary features are present (5, 6).

The incidence of BBS ranges from nearly 1 in 125,000 in North America and Europe to 1 in 17,000 in the Bedouins of Kuwait, and 1 in 3700 in the Faroe Islands. The higher incidence is observed in geographically isolated or highly consanguineous populations (9-12). 

To date, causative mutations in 19 genes, including *BBS1*,* BBS10*,* BBS2*,* BBS9*,* MKKS*,* BBS12*,* MKS1*,* BBS4*,* BBS7*,* TTC8*,* ARL6*,* BBIP1*,* BBS5*,* CEP290*,* IFT27*,* LZTFL1*,* SDCCAG8*,* TRIM32*, and* WDPCP* (in order of attribution) can cause BBS, which can account for the causality in about 80% of the examined cases (3, 8). These genes consist of over 200 coding exons, which makes it a laborious time-consuming job to analyze them by conventional PCR-Sanger sequencing method. Next-generation sequencing (NGS) has begun to be widely used for the analysis of genetically heterogeneous monogenic diseases, such as BBS. Targeted multi-gene NGS panels have provided a faster and more efficient approach regarding coverage, costs, time, and handling (13).

In this study, we investigated two Iranian unrelated clinically diagnosed BBS patients, using a targeted NGS panel consisting of 18 known BBS genes.

## Materials & methods

**Patients** **and sampling:**

**Figure 1:**
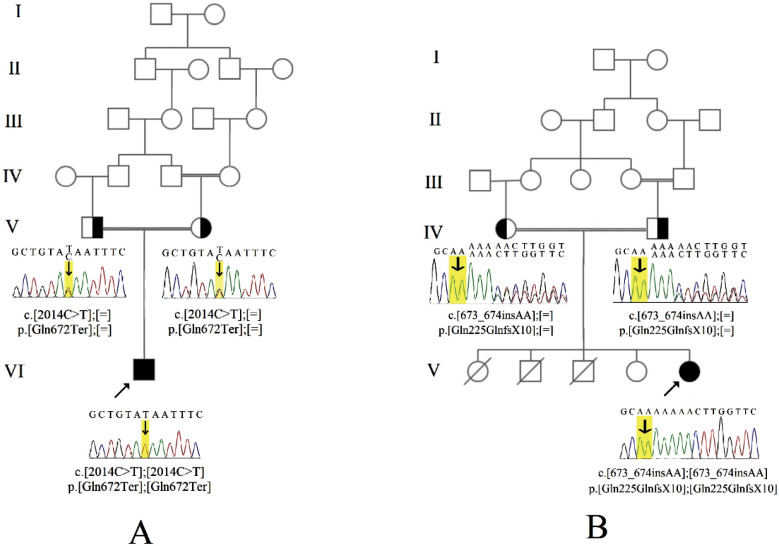
Pedigrees A: Patient 1, B: Patient 2 and the result of Sanger sequencing validation of the detected variants

We investigated two unrelated clinically diagnosed BBS patients who were the children of the first-cousin consanguineous marriage. The pedigrees are illustrated in Figure 1. The patients were originally from two geographically distinct regions in Iran. Patient one had Lur ethnicity and was from central west of Iran. Patient two had Turk ethnicity and was from the north-west of Iran. A comprehensive clinical examination was performed by the pediatrician. 

This study was approved by the Research Ethics Committee of Tehran University of Medical Sciences, Tehran, Iran. Written informed consent was obtained from the parents of the patients and peripheral blood samples were collected from the patients and their unaffected parents.

**Genetic analysis:**

**Targeted NGS:**

The DNA isolation was performed from the whole blood using the GeneAll® Exgene™ Blood SV DNA purification kit according to the manufacturer’s instructions. Target region capturing was performed with a custom-designed Nimblegen chip for coding regions and the exon-intron boundaries of 18 BBS-related genes (*BBS1*, *BBS2*, *ARL6*, *BBS4*, *BBS5*, *MKKS*, *BBS7*, *TTC8*, *BBS9*, *BBS10*, *TRIM32*, *BBS12*, *MKS1*, *CEP290*, *SDCCAG8*, *WDPCP*, *TMEM67*, and *LZTFL1*). Next-generation sequencing was performed on an Illumina HiSeq 2500 NGS platform (Illumina Inc., San Diego, CA, USA) provided by Beijing Genomics Institute (BGI, Shenzhen, Guangdong, China).

**Variant analysis:**

The detected variants were looked up in literature, population databases [Exome Aggregation Consortium (ExAC), 1000 Genomes Project (1000G), and dbSNP], disease databases [ClinVar, Online Mendelian Inheritance in Man (OMIM), Human Gene Mutation Database (HGMD)], and sequence databases (NCBI Genome, Ensembl) for any previously reported record. The variants were also analyzed by several computational (*in silico*) tools (Mutation taster, CADD, phyloP, phastCons) to determine the nucleotide and amino acid conservation status and effect of the sequence variant at the nucleotide and amino acid level, including determination of the effect of the variant on the primary and alternative gene transcripts and other genomic elements, as well as the potential impact of the variant on the protein (15). Furthermore, the variants were checked to see if they are located in any mutational hot spot and/or critical and well-established functional domain (14). The protein domains, in which the variants were located, were also obtained using Pfam and Interpro databases. 

The variant identified by NGS analysis in each patient was confirmed by Sanger sequencing (ABI3700 automated sequencer, Pishgam Biotech Company, Tehran, Iran). Segregation analysis of the detected variants was performed in each pedigree by polymerase chain reaction (PCR) followed by Sanger sequencing. Table 1 shows the primers and conditions used for amplifying exons 7 and 19 of *BBS9*. For exon 19, the sequencing was done using universal primers; M13F (tagged to forward primer), or M13F-pUC(-40) (tagged to reverse primer). Exon 7 was sequenced by a forward PCR primer.

## Results

The clinical characteristics of the identified variants are summarized in Table 2.

Table 3 represents the NGS parameters of the test in each patient. One homozygous null variant was detected in each patient in *BBS9* (Table 3). A stop-gained nonsense variant was detected in patient one and a frame-shift caused by a two-base-pair insertion was detected in patient two.

The detected variants were not previously reported in any of the population databases (ExAC, 1000G, dbSNP). Characteristics of the identified variants and *in* *silico* analysis results are provided in Table 4. 

The results of Sanger sequencing validation of the detected variants in the patients and the sequencing results of the same point in their parents are demonstrated in Figure 1.

**Table 1:** Primers and PCR conditions used to amplify exons 7 and 19 of *BBS9* (universal tags are underlined)

Exon Number	5ʹ-3ʹ primer sequences	Product size (bp)	Touch down annealing temperature for PCR (°C)
7	TTGGTGTTTAGGTGCTAGATCT	566	72 to 60
	AACCAACTCAATTCTTAGGTCA		
19	GTAAAACGACGGCCAGTTGATCTGTTTTTCCTCCAGTGATAC	491	72 to 60
	GTTTTCCCAGTCACGACACTGGAAATGAGTACCTTATCTTGG		

**Table 2:** The clinical characteristics

**Clinical Characteristics**	**Patient 1**	**Patient 2**
Gender	Male	Female
Ethnicity (geographic region in Iran)	Lur (Central west of Iran)	Turk (North west of Iran)
Age	9	13
Height	137 cm	140 cm
Weight	34	67
Parents’ consanguinity	Yes (first cousins)	Yes (first cousins)
**Major BBS features**		
Retinitis Pigmentosa	Yes	Yes
Postaxial polydactyly	Yes (Bilateral upper and right lower limb) (surgical reconstruction)	Yes (Right upper limb) (surgical reconstruction)
Truncal obesity	Yes	Yes
Learning disabilities	Yes	Yes
Hypogonadism (in males) or genital abnormalities (in females)	Yes (Hypogenitalism, microphallus)	Yes (Hypogonadism)
Renal anomalies	No	No
**Secondary Features**		
Speech delay	Yes	Yes
Developmental delay	Yes	Yes
Behavioral abnormalities	?	?
Eye abnormalities / Craniofacial dysmorphism	Yes (strabismus, long Eyelashes, epicanthal folds, almond-shaped palpebral fissures, down-slanting palpebral fissures, bi-temporal narrowing, Palatal pits)	Yes (strabismus, down-slanting palpebral fissures, bi-temporal narrowing)
Brachydactyly/syndactyly	Yes/Yes	Yes/Yes
Ataxia/poor coordination	No/No	Yes/Yes
Mild hypertonia (especially lower limbs)	No	?
Diabetes mellitus	No	No
Oro-dental abnormalities	Yes (High arched palate)	No
Cardiovascular anomalies	No	Yes (cardiomegaly in CXR and severe right ventricular dysfunction in echocardiogram)
Hepatic involvement	No	No
Hirschsprung disease	No	No
Anosmia	No	No
Other findings	Simian crease Head circumference: 56 cm Persistent fetal pads Nail-hypoplasia Normal metabolic test results Normal male karyotype Normal brain MRI Bifid Scrotum Bilateral 5th finger clinodactyly Sacral pit Clubfeet Simian crease Head circumference: 52 cm Persistent fetal pads Nail-hypoplasia Normal female karyotype History of three siblings’ death at ages of 26 days (male), 3 days (female), and 3days (male) old with cyanosis following breast-feeding, and an older alive sister Left sided weakness in movements from the birth Cyanotic and weak cry at birth Breath-holding spells Drop attacks History of retinitis pigmentosa in her older sister Brachycephaly Flat occiput Hyperpigmentation of cervical skin Tapered fingers Small hands Obesity stretch marks Mild dyspnea	

? : Not evaluated

**Table 3:** NGS parameters

Parameters	Patient 1	Patient 2
Coverage of target region	100.00%	100.00%
Average depth of target region (X）	288.24	235.93
Proportion of target region with sequencing depth more than 30X	99.64%	99.46%

**Figure 2:**
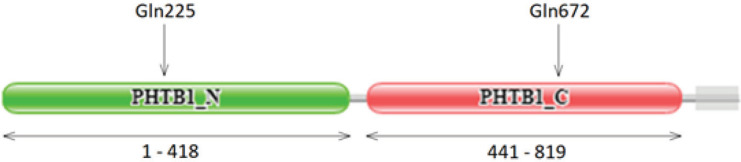
Domains of PTHB1 protein and the amino acid position of the truncation (Gln225 in patient 1 and Gln672 in patient 2)

**Table 4:** Characteristics of the identified variants in BBS9 (RefSeq: NM_198428)

Patient	Position (GRCh37)	cDNA	Protein	Exon	Type of variant	domain	CADD PHRED score	Mutation taster	phastCons	phyloP	ExAC freq.	1000G freq.	dbSNP	Parental origin
1	chr7:33427655	c.2014C>T	p.Gln672Ter	19/23	NS	Ct	45	DC	1	5.053	-	-	-	Maternal and paternal
2	chr7:33303957-33303958	c.673_674insAA	p.Gln225GlnfsX10	7/23	FS	Nt	35	DC	nt*673: 1 nt 674: 1	nt 673: 4.707 nt 674: 2.392	-	-	-	Maternal and paternal

NS: Nonsense, FS: Frameshift, Ct: C-terminus domain, Nt: N-terminus domain, DC: Disease causing, Freq.: Frequency

## Discussion

We performed a targeted NGS panel, covering 18 BBS genes, in two patients with a clinical diagnosis of BBS. The two detected *BBS9* variants, c.2014C>T, p.Gln672Ter in patient one and c.673_674insAA, p.Gln225GlnfsX10 in patient two, were confirmed with Sanger sequencing and were segregated in the pedigree in an autosomal recessive pattern. These variants have not been previously reported for their pathogenicity. Both alterations have been expected to result in a truncated protein product and activate the nonsense-mediated decay (NMD) mechanism. NMD acts as a surveillance system and protects the cell against the toxic effect of a protein with a premature termination codon (PTC). To do so, the PTC should be located in the NMD target region (16, 17). Here, both detected variants were located in activating NMD region. In addition, both variations fall into functional domains of the protein, C-terminus domain, and N-terminus domain, which are both required for ciliogenesis (Figure 2). Therefore, the detected variations are expected to have a major effect on the protein's function (if any protein is produced) and finally on BBSome (18, 19). It can be inferred that both variants lead to no gene product and act as null variants. Previous studies have confirmed that nonsense and frame-shift mutations in BBS-related genes are among the mechanisms resulting in BBS (20, 21). 

*BBS9* gene is also associated with other disorders; the gene has been a candidate for Wilms' tumor (22) and also has a strong association with primary ovarian insufficiency (23). *BBS9* product is a core component of BBSome, a complex with a key role in ciliogenesis and also intraflagellar transport (IFT) assembly (24).

The two detected variants in this study were absent in ExAC and 1000G population databases. Computational analysis by multiple tools supports the disease-causing effect of both variants. According to the American College of Medical Genetics and Genomics (ACMG) guidelines, both identified variants are classified as "pathogenic" variants. The clinical diagnosis of the disease was confirmed and the health care provider could use the molecular testing information in clinical decision-making. Furthermore, variants classified as “pathogenic” according to ACMG guidelines, can be used in pre-implantation genetic testing (PGD) and prenatal diagnosis (PND) in the future pregnancies of the pedigree. 

To date, causative mutations in 19 genes, including *BBS1*,* BBS10*,* BBS2*,* BBS9*,* MKKS*,* BBS12*,* MKS1*,* BBS4*,* BBS7*,* TTC8*,* ARL6*,* BBIP1*,* BBS5*,* CEP290*,* IFT27*,* LZTFL1*,* SDCCAG8*,* TRIM32*, and* WDPCP* (in order of attribution) have been identified to cause BBS, which account for the causality in about 80% of the examined cases (3, 8).

Bardet-Biedl syndrome is a genetic disorder with a pleiotropic characteristic. Affected patients show a wide range of phenotypic features (6). Intrafamilial variability has been reported also (25, 26). Although two patients in this study were from different families, they were very similar in major and minor BBS phenotypic features, which can be due to having truncating mutations in the same gene resulting in the same type of BBS, i.e. type 9. Patient two had some cardiovascular anomalies, which were absent in patient one. They were also different in some facial features. Patient two showed ataxia but there was no sign of it in patient one. Both patients had normal hearing; however, there are reports of hearing problems in patients with *BBS9* mutations (8). The minor phenotypic differences may partly be explained by their different mutations, patients’ gender, age, and the role of the modifying genes.

BBS is known as an autosomal recessive disorder; however, some pieces of evidence propose an oligogenic pattern of inheritance in some cases. *BBS9* mutations could play the role of the "third" allele along with a homozygote mutation in another gene resulting in an oligogenic inheritance (25); however, in the current study, it was not the case; the detected mutations were homozygous and showed strong evidence to be considered as the causative variants in these cases. Yet the patients had no known mutations or unknown variants suspicious to be pathogenic in other BBS genes examined. 

Several reviews have estimated a ~ 6% contribution of *BBS9* to the disease causality in BBS patients (6, 8). Interestingly, we visited these two patients with mutations in *BBS9* in a short period of time while referring to the same clinical center. A previous study on Iranian patients also showed a comparatively high (14%) contribution for *BBS9* (27). However, a study on Turkish patients (neighbor country of Iran) found a 6.6% casualty for *BBS9* (28). Therefore, a study with large sample size is needed to clarify any possible significant deviation of mutational load for each BBS gene in Iranian patients.

Generally, studies with a large sample size have not been performed on Iranian BBS patients yet. Because of a high rate of consanguineous marriages in the Iranian population, novel and family-specific mutations are typically expected to be detected in the case studies of the genetic hereditary diseases in Iran, similar to the current study. 

**In Conclusion,** our study reported two novel variations in *BBS9*, a nonsense variant in exon 19 and a frame-shift variant in exon 7, in two unrelated Iranian families with an affected member in each. The variants were categorized as “pathogenic”. The clinical diagnosis was confirmed and the families were referred for comprehensive genetic counseling before PGD and PND in their future pregnancies. 

## Author’s Contribution

These authors contributed equally to this work and are considered as co-first-authors.

## Conflict of interest

The authors report no conflict of interest
